# Case report: A rare case of anomalous origin of the left coronary artery from the pulmonary artery accompanied with unilateral absence of pulmonary artery in an adult patient

**DOI:** 10.3389/fcvm.2023.1160893

**Published:** 2023-04-20

**Authors:** Dong Yi, Juan Xia, Xiang Zhou, Chengwei Liu, Li Liu, Hua Yan, Xiaojing Ma

**Affiliations:** ^1^ Department of Cardiology, Wuhan Asia Heart Hospital, Wuhan, China; ^2^ Department of Echocardiography, Wuhan Asia Heart Hospital, Wuhan, China; ^3^ Department of Cardiac Surgery, Wuhan Asia Heart Hospital, Wuhan, China

**Keywords:** anomalous origin, left coronary artery, pulmonary artery, echocardiography, coronary angiography, multidetector computed tomographic angiogram

## Abstract

Both the anomalous origin of the left coronary artery from the pulmonary artery (ALCAPA) and unilateral absence of the pulmonary artery (UAPA) are rare congenital malformations, ALCAPA accompanied with UAPA is extremely rare. Here, we reported a middle-aged man admitted to our department for evaluation of chest pain during exercise. Physical examination and lab tests did not unveil obvious abnormality; however, transthoracic echocardiogram (TTE) revealed multivessel myocardial collateral blood flow signals in the left ventricular wall and ventricular septum, a shunting flow from the left coronary artery into the pulmonary artery and dilated right coronary artery (RCA), which supported but did not confirm the diagnosis of ALCAPA. Coronary angiography (CAG) showed an absent left coronary ostium and a dilated RCA, with extensive collaterals supplying the left coronary system. Multidetector computed tomography angiography (MDCTA) was then performed and revealed the anomalous origin of the left main coronary artery (LMCA) arising from the pulmonary artery, and it incidentally unveiled another rare congenital malformation of UAPA. The patient underwent surgical correction of ALCAPA by reimplantation of the LMCA to the aorta, without surgical treatment of UAPA. The patient had been in good clinical condition and remained angina free with good exercise tolerance during follow-up (∼6 months so far). In this case, we discussed the diagnostic value of TTE, CAG, and MDCTA on rare abnormalities as ALCAPA and UAPA. We highlighted the role of multiple non-invasive imaging modalities in diagnosing rare causes of angina in adult patients, and the importance of careful examination in avoiding misdiagnosis. To our best knowledge, this is the first report of ALCAPA accompanied with UAPA in an adult patient.

## Introduction

Chest pain is one of the most common complaints of patients seen in the ambulatory practice, and cardiac ischemia caused by atherosclerotic coronary heart disease is one of the most common etiologies. In the approach to the patient who has chest pain, a detailed history, physical examination, and some auxiliary examinations, such as electrocardiogram (ECG) and transthoracic echocardiogram (TTE), can help physicians identify the cause. However, it is very challenging for the clinicians to evaluate chest pain from rare causes, such as anomalous origin of the left coronary artery from the pulmonary artery (ALCAPA). ALCAPA is a very rare congenital heart anomaly, comprising approximately 0.25%–0.5% of all congenital heart anomalies (1, 2). The mortality rate is nearly 90% in affected infants if untreated, but patients can live to adults if intracoronary collaterals were well-established (3, 4). The clinical manifestations in adult patients with ALCAPA varies from angina, dyspnea, palpitations, and ventricular arrhythmia to syncope, even sudden death, while less than 15% of the patients remained asymptomatic (4–6). Surgical intervention with direct reimplantation of the ALCAPA into the aorta is the standard treatment option (2). Early diagnosis and timely surgical intervention are imperative for patients with ALCAPA.

Unilateral absence of the pulmonary artery (UAPA) is another rare congenital malformation with a prevalence of 0.0005%, which is often associated with other congenital cardiac defects such as aortic coarctation, tetralogy of Fallot, atrial septal defect, patent ductus arteriosus, truncus arteriosus, right aortic arch, and pulmonary atresia (7–9). UAPA is more common on the right side and usually asymptomatic until adulthood if absence of other associated cardiac malformation (8, 9). Adults with isolated UAPA can present with recurrent respiratory infections, dyspnea, exercise intolerance, chest pain, and hemoptysis whereas hemoptysis is the commonest manifestation in adults (8–10). Currently, there is no consensus on how to effectively manage adult patients with UAPA. There are a few treatment options available for those with isolated UAPA, including selective embolization of the collateral artery, pneumonectomy, and antipulmonary hypertension medication (8).

Herein, we report an extremely rare case of ALCAPA accompanied with UAPA who presented to the hospital with angina. The diagnosis was difficult and complicated. Multiple imaging methods, namely, TTE, multidetector computed tomography angiography (MDCTA), and invasive coronary angiography (CAG), were used to reach the diagnosis. Surgical correction was performed and confirmed the diagnosis. In this case, we discussed the diagnostic findings of TTE, CAG, and MDCTA and emphasized the role of multiple imaging modalities in diagnosing rare causes of angina in adult patients. To our best knowledge, this is the first report of ALCAPA accompanied with UAPA in an adult patient.

## Case presentation

**Figure 1:**
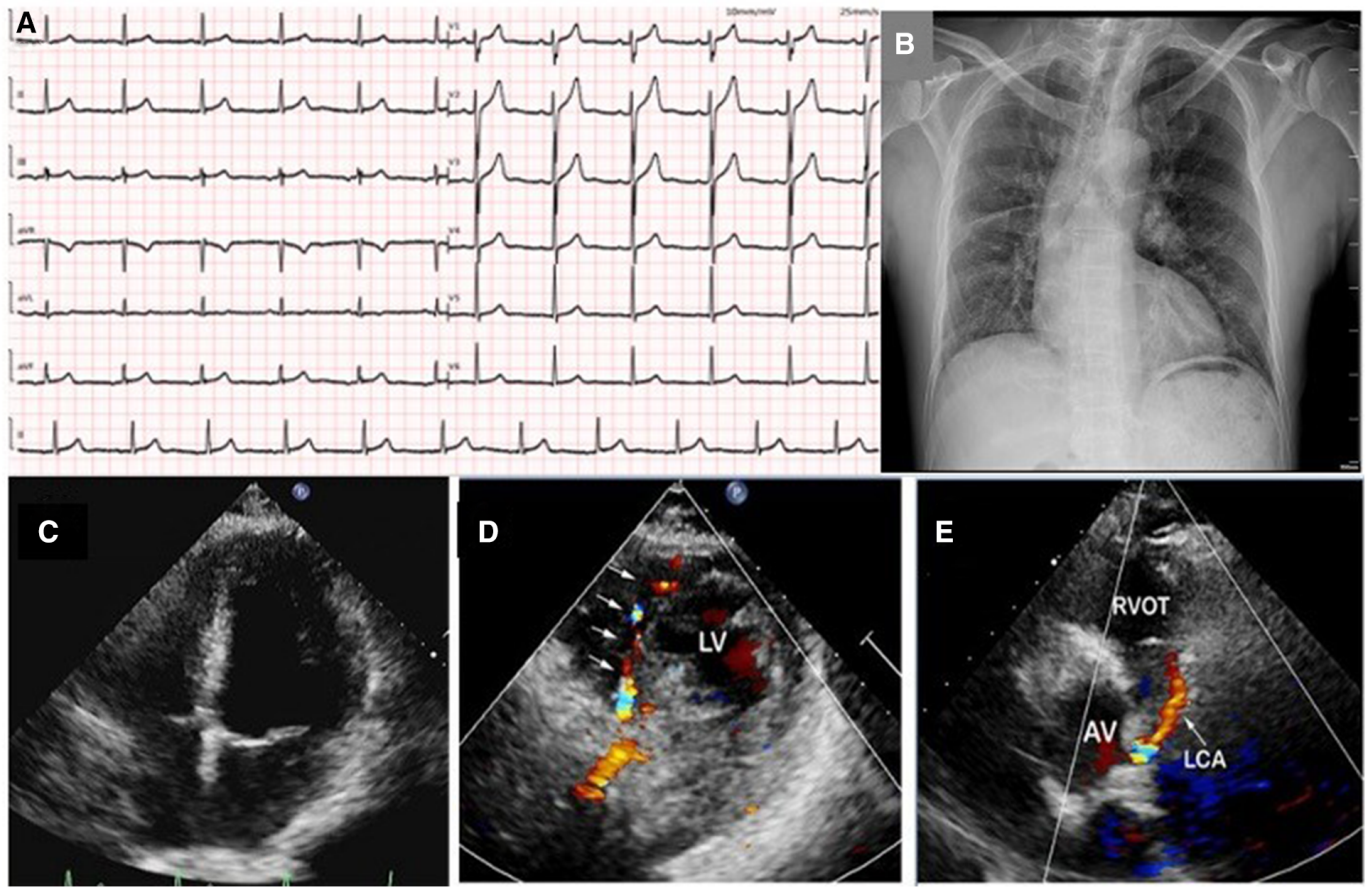
A 12-lead ECG illustrated normal sinus rhythm without ST-segment and T-wave changes (**A**); chest x-ray showed scoliosis, cardiac enlargement and briefly normal vascular markings on both sides, along with rightward mediastinal shift (**B**); transthoracic echocardiography revealed normal size of four chambers, left ventricular, and valvular function (**C**); color Doppler in parasternal short-axis view showed abundant collateral blood signals within the ventricular septum (**D**); an abnormal continuous blood flow directed into the pulmonary artery was also observed (**E**). LV, left ventrium; RVOT, right ventricular outflow tract; AV, aortic valve; LCA, left coronary artery.

A 49-year-old man with no previous medical history presented to our hospital with complaint of chest pain, palpitations, and diaphoresis during exercise for 3 months. The chest pain was able to be relieved by rest and nitrates. Upon admission, physical examination revealed normal range of blood pressure (135/74 mmHg), heart rate (78 beats/min), respiratory rate (18/min), and oxygen saturation level was 99% (room air). He denied history of hypertension, diabetes, and family history of heart diseases. Personal habits included smoking (half pack/day) and drinking. Cardiac and pulmonary examination revealed normal heart sounds without murmurs and clear lungs. Laboratory tests indicated that complete blood count with differential, comprehensive metabolic panel, renal function, troponin, N terminal pro-brain natriuretic peptide (NT-proBNP), and several tumor markers were within normal limit. A 12-lead ECG illustrated normal sinus rhythm without ST-segment and T-wave changes (Figure 1A). Chest x-ray showed scoliosis, cardiac enlargement, and briefly normal vascular markings on both sides, along with a rightward mediastinal shift (Figure 1B).

TTE revealed normal size of chambers and left ventricular systolic function with left ventricular ejection fraction 60%. No abnormal wall motion and valvular regurgitation were observed. However, multivessel myocardial collateral blood flow signals at the left ventricular wall and ventricular septum were observed by color Doppler flow imaging (CDFI). At parasternal short-axis view, a red shunting flow seemingly from the left coronary artery (LCA) into the pulmonary artery (PA) was seen by CDFI, and a continuous shunt following the path of this vessel, which appeared as low velocity with pronounced diastolic phase on pulsed Doppler was also detected (Figures 1C–E and Supplementary Videos S1A,S1B). The ostiums of the left and right coronary artery (RCA) were not clearly visualized with TTE in this patient. At the same time, the main pulmonary artery exhibits normal blood flow and pressure.

**Figure 2:**
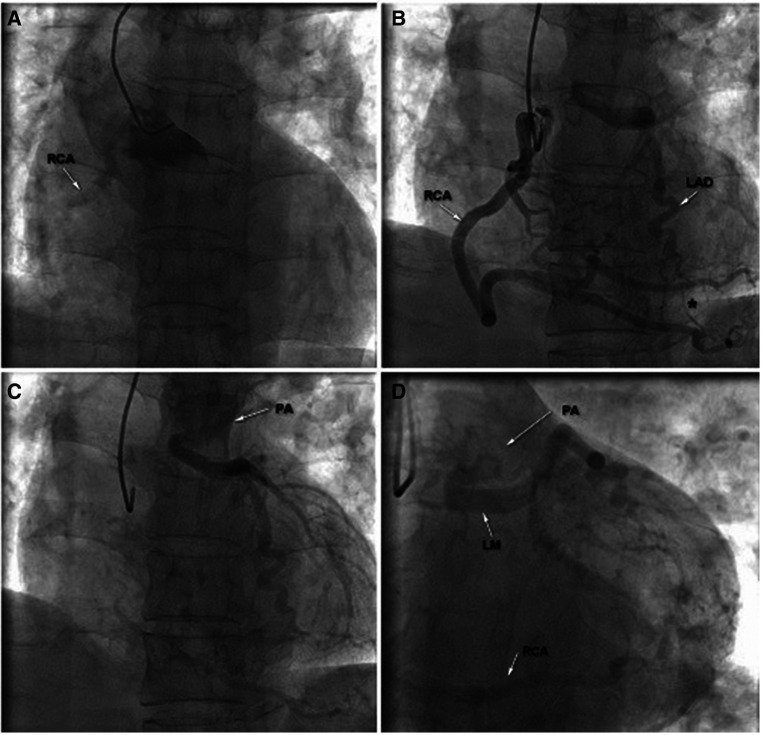
Transradial coronary angiography was performed; however, multiple attempts failed to engage the left main coronary artery (LM) (**A**); right coronary angiography revealed an extremely large and tortuous RCA arising from the RCC with significant collateral flow from RCA to LCA (**B**). The blood flow terminated in the pulmonary artery through the LCA and delayed enhanced-imaging demonstrated the LCA filling via extensive intercoronary collateral circulation, which supported the diagnosis of ALCAPA (**C,D**). LA, left main coronary artery; LAD, left anterior descending; PA, pulmonary artery; RCA, right coronary artery.

The patient was then transferred to the cardiac catheterization laboratory for further evaluation. Multiple attempts failed to engage the left main coronary artery (LMCA), but the transradial CAG revealed an extremely large and tortuous RCA arising from the right coronary cusp (RCC) with significant collateral flow from RCA to LCA. The blood flow terminated in the pulmonary artery through the LCA, and delayed enhanced-imaging demonstrated the LCA filling via extensive intercoronary collateral circulation, which supported the diagnosis of ALCAPA (Figures 2A–D and Supplementary Videos S2A,S2B). Surgical correction for ALCAPA was planned.

**Figure 3:**
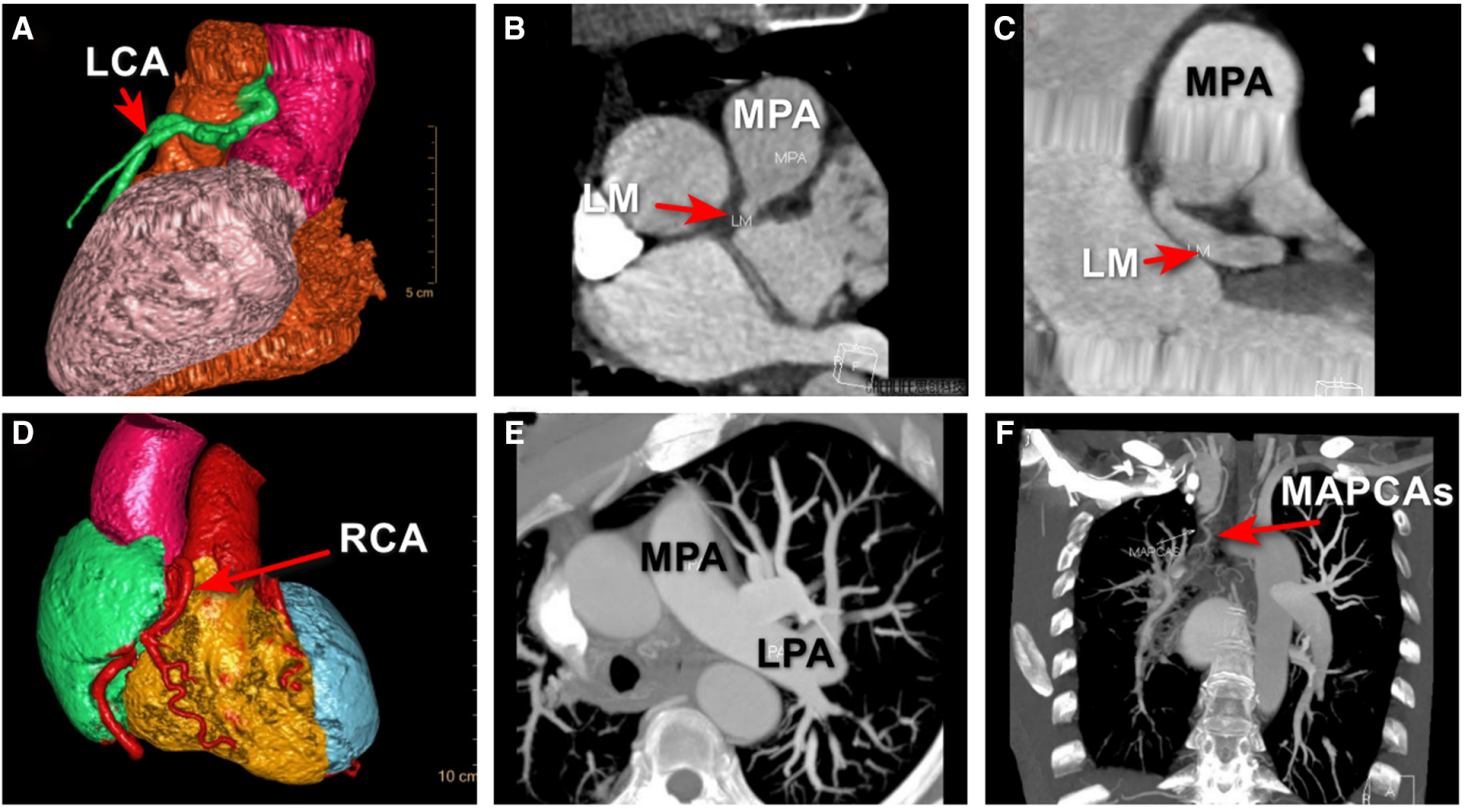
MDCTA revealed that the LCA originated from the right side of main pulmonary trunk and then bifurcated into the left anterior descending artery and the left circumflex artery (**A–C**); MDCTA showed the compensatory dilated and tortuous RCA (**D**); an UAPA was incidentally revealed by MDCTA (**E**); and major aortopulmonary collateral artery was shown (**F**), which was missed with TTE and CAG. ALCAPA, anomalous origin of the left coronary artery from the pulmonary artery; LCA, left coronary artery; LM, left main coronary artery; LPA, left pulmonary artery; MAPCAs, major aortopulmonary collateral arteries; MPA, main pulmonary artery; UAPA, unilateral absence of pulmonary artery.

To better assess the significance of this anomaly and provide detailed information about this anomaly for the surgeon, MDCTA was ordered and clearly revealed that the LCA originated from the pulmonary artery and then bifurcated into the left anterior descending artery and the left circumflex artery. The RCA was diffusely enlarged and tortuous, arising from the right coronary sinus as normal, which further confirmed the diagnosis of ALCAPA (Figures 3A–C).

However, a unilateral absence of the right pulmonary artery was incidentally revealed by MDCTA, which was missed with TTE and CAG (Figures 3D–F). Major aortopulmonary collateral artery was shown (Figure 3F). Through a more comprehensive analysis of the MDCTA images, we have confirmed that the circulation supplying the right lung originates from the right subclavian artery, with a potential contribution from a collateral bronchial artery and that there was no evidence of collateral circulation within the left lung tissue. At the same time, upon further review of the MDCT images, we basically excluded certain diseases that have similar imaging characteristics, such as pulmonary atresia, pulmonary artery arising from the subclavian or innominate artery, and pulmonary artery disconnection.

The patient received surgical correction of this anomaly by reimplantation of the LMCA to the aorta. During the procedure, origin of the LCA from the PA trunk, a dilated and tortuous RCA from the RCC, extensive intercoronary collaterals, and UAPA were conﬁrmed. The patient was discharged from the hospital 2 weeks after surgery without complications. At his last (6 months) follow-up, the patient had been in good clinical condition and remained angina-free with good exercise tolerance.

## Discussion and conclusions

ALCAPA is an unusual congenital heart defect which affects approximately one in 300,000 live births and accounts for 0.26% of all congenital heart diseases (1, 2). Anatomically, LCA originates from PA instead of the aorta, leading to a “coronary steal” phenomenon due to lower pulmonary artery pressure. The blood flowed away from the myocardial vessels to the pulmonary artery resulting in a left to right shunting of the blood through ventricular septum or ventricular wall; therefore, most of the patients presented symptoms of myocardial ischemia. Without surgical intervention, most patients die in infancy (nearly 90%) (2, 3). Only patients with adequate collateral circulations between the RCA to LCA may survive beyond infancy (3, 4). ALCAPA presentation in adults is rare, and around 90% of adults may suffer from sudden death at a mean age of 35 (11).

The diagnosis of ALCAPA is complicated, non-invasive techniques, such as TTE, play an important role in diagnosis and differential diagnosis. The typical echocardiographic features of ALCAPA include the following: (1) the ostium of the LCA originates from the pulmonary artery rather than aorta in parasternal aortic root short-axis view; (2) multivessel myocardial collateral blood flow signals in the left ventricular wall and ventricular septum by CDFI; (3) the retrograde blood flow in the abnormal vessel, which indicating left-to-right shunting from RCA via LCA to main pulmonary artery through highly developed collateral vessels; and (4) proximal dilated and tortuous RCA. The major differentiation included RCA to pulmonary artery fistula and multiple muscular ventricular septal defects (12, 13). CAG was usually performed to confirm the diagnosis when ALCAPA was suspected by echocardiography, which can provide details of origination of coronary, running route (pathway), and presence/absence of diseased coronary artery. MDCTA has been increasingly used to assess the coronary anatomy in recent years with very good diagnostic accuracy and is considered as a reliable tool to evaluate the coronary anatomy and establish the diagnosis (14, 15). Once diagnosed, surgical correction is highly recommended for all of the patients with ALCAPA (3–5), and the coronary artery reimplantation from PA to the aorta was the most used procedure for the cardiac surgeon. The long-term outcome of the procedure was usually favorable.

UAPA is also a rare congenital anomaly, which is the absence of the left or right pulmonary artery caused by involution of the proximal sixth aortic arch. In most patients, UAPA are diagnosed in infancy or childhood due to symptoms related to cardiovascular abnormalities (7, 8). In adult patients, common presenting symptoms include recurrent respiratory infections, dyspnea, exercise intolerance, hemoptysis, and chest pain (7–9). However, approximately 30% of patients with UAPA were asymptomatic, and the diagnosis was incidental (9). Hemoptysis is caused by excessive collateral circulation, may be self-limiting or massive, and is life threatening (9). Although TTE was usually used as a primary method for diagnosing UAPA, it often undiagnosed this rare anomaly due to absence of symptoms. In our patient, the UAPA was diagnosed incidentally by MDCTA, which was initially used to reveal the detail of ALCAPA before surgery. The therapeutic approach for isolated UAPA should be based on symptoms of the patient, PA anatomy, associated cardiovascular anomalies, and aortopulmonary collaterals and pulmonary hypertension. No treatment is required in patients without any evidence of cardiopulmonary dysfunction (8).

To our best knowledge, this is the first report of ALCAPA accompanied with UAPA in an adult. Early recognition and management of both ALCAPA and UAPA are very important. But the diagnosis is difficult, especially for those asymptomatic adult patients with normal ECG and echocardiographic examination. Dilated and tortuous RCA, anomalous origin of the LCA and abnormal pulmonary cavity shunt are often overlooked by the echocardiographer. However, the abundant collateral circulation in the interventricular septum was easily noticed, which was an important clue, prompting the echocardiographer for further investigation. CAG and MDCTA are both superior to echocardiography in showing the origin of the coronary artery, running route, positional information, and absence/presence of other anomalies. In this report, we highlight the important role of echocardiography as the primary method for diagnosis of ALCAPA and CAG and MDCTA as necessary addition to confirm diagnosis and finding other abnormalities.

## Data Availability

The original contributions presented in the study are included in the article/**Supplementary Material**. Further inquiries can be directed to the corresponding authors.

## Supplementary material

The Supplementary Material for this article can be found online https://www.frontiersin.org/articles/10.3389/fcvm.2023.1160893/full#supplementary-material.
